# Effects of Normative Aging on Eye Movements during Reading

**DOI:** 10.3390/vision4010007

**Published:** 2020-01-14

**Authors:** Kevin B. Paterson, Victoria A. McGowan, Kayleigh L. Warrington, Lin Li, Sha Li, Fang Xie, Min Chang, Sainan Zhao, Ascensión Pagán, Sarah J. White, Jingxin Wang

**Affiliations:** 1 Department of Neuroscience, Psychology and Behaviour, University of Leicester, Leicester LE1 7RH, UK; vm88@leicester.ac.uk (V.A.M.); klw53@leicester.ac.uk (K.L.W.); appc1@leicester.ac.uk (A.P.); sjw92@leicester.ac.uk (S.J.W.); 2 Faculty of Psychology, Tianjin Normal University, Tianjin 300387, China; linpsy@outlook.com (L.L.); xftjsf@126.com (F.X.); tjsfchangmin@126.com (M.C.); sainanpsy@163.com (S.Z.); wjxpsy@126.com (J.W.); 3 School of Psychology, Fujian Normal University, Fuzhou 350117, China; llesq@outlook.com

**Keywords:** cognitive aging, eye movements during reading, alphabetic reading, Chinese reading

## Abstract

Substantial progress has been made in understanding the mostly detrimental effects of normative aging on eye movements during reading. This article provides a review of research on aging effects on eye movements during reading for different writing systems (i.e., alphabetic systems like English compared to non-alphabetic systems like Chinese), focused on appraising the importance of visual and cognitive factors, considering key methodological issues, and identifying vital questions that need to be addressed and topics for further investigation.

## 1. Introduction

When people read, their eyes make rapid movements (called saccades), separated by brief fixational pauses during which visual information is obtained from text (for reviews, [1,2,3]). These eye movements are closely coordinated with processes of perception, attention, language processing, and memory. This enables skilled readers to recognize words very rapidly as their eyes move across a text, at a rate of about 3 to 5 words per second, and for the meaning of these words to be integrated with the reader’s evolving interpretation of the text. A substantial body of research over the past 40 years has focused on understanding how this feat is accomplished by skilled adult readers [2,3]. More recent research has also begun to investigate how this capability develops in beginner readers [4,5], as well as how it changes across the lifespan.

With the present review, we focus on the effects of normative, healthy aging. Being able to read well is important for individuals to function effectively in most modern societies, and to accomplish everyday tasks that enable them to live independently. However, changes in the visual and cognitive systems that occur naturally in healthy aging are likely to affect the ability of older adults to read effectively. Such changes begin in middle age but are especially marked in later adulthood (from about 65 years of age onwards; for reviews, see [6,7,8,9,10]). At the level of the eye, these include narrowing of the pupil (pupillary miosis) and increased opacity of the lens, reducing the passage of light through the eye, while a loss of elasticity of the lens limits the ability to focus on near objects (presbyopia). As a result, less light falls on the retina and the image it creates is blurred. These optical changes combine with retinal and neural changes, including loss of retinal photoreceptive cells (rods and cones), which limit visual sensitivity, and reductions in axons in the optic nerve and neurons in the visual cortex, which affect the transmission and processing of visual information.

Together, these changes decrease the efficiency of visual processing. Functionally, they are associated with lower acuity and reduced ability to perceive fine visual detail, especially in low light or when contrast is reduced [11,12,13,14]. This can contribute to the experience of visual blur [15], as well as problems reading fine-print [16] or in dim lighting conditions [17]. Reductions in visual sensitivity are also greater outside central vision [18] so that older adults see detail more poorly in peripheral vision, which may help explain why they appear to have a narrower field of effective vision [19,20]. Reductions in visual sensitivity are experienced even when vision is corrected optically. Consequently, even older adults who wear their habituation optical correction (glasses, contact lenses) will experience degradation in visual input and process visual information more slowly and with greater error. Age-related declines in attentional and motor control may also affect the efficiency of eye movement control. Non-reading studies consistently show that aging is associated with an increase in the time to initiate a saccade in response to a visual target [21,22,23,24,25,26,27], and the velocity of the resulting movement [28,29]. However, accuracy in saccade-targeting [23,25,30,31] and fixation stability [32] appear to be spared.

Finally, declines in memory capabilities may affect the retrieval of information from memory and short-term storage and manipulation of linguistic information in working memory [33,34,35,36]. It seems likely that effects on retrieval will impair processes of word recognition, while working memory deficits may impair comprehension [37,38]. However, while aging produces deterioration in elements of what often is described as fluid intelligence (e.g., memory, reasoning, problem-solving [9,39]), crystalized intelligence, which includes knowledge gained from a lifetime of experience of reading and exposure to language, appears stable across adulthood and only shows declines in very old age [8]. Crucially, crystalized knowledge may offset deficits in fluid intelligence and have a preservative effect on reading. For instance, even though older people can have difficulty bringing words to mind, they tend to outperform younger adults on vocabulary tests, due to superior knowledge of words gained from their greater reading experience [40,41]. Note, however, that an alternative account postulates that the slowdown in lexical access in older age is because of difficulty discriminating individual words in the mental lexicon as a result of a lifetime of accumulation of lexical items rather than cognitive decline [42,43]. Consequently, while research inevitably focuses on loss in abilities, it is important to remember that some losses may be offset by superior skills elsewhere, while other abilities may be highly resilient. Older adults may also use adaptive strategies to compensate for loss of abilities, including changing how they allocate processing resources [44,45,46].

With the present review, we focus on effects of normative aging on eye movements in reading. This is because measures of eye movements provide a highly naturalistic means of studying the process of reading, as participants read text presented normally at their own pace. A further advantage comes from the close yoking between eye movements and cognition, such that where a reader is looking is informative about what they currently are processing, while the length of time they look in this location is sensitive to any processing difficulty they are experiencing [2,3]. However, it is important to acknowledge valuable research that has used other methods to gain insight into aging effects on word recognition [47,48,49,50,51], sentence and discourse comprehension [52], and the neural correlates of these processes [53,54,55,56,57,58].

## 2. Aging Effects on the Perceptual Span

A first issue concerns whether aging limits the amount of linguistic information acquired on each fixational pause. Readers make eye movements because they cannot process an entire line of text on a single glance and so make multiple glances to obtain this information in discrete chunks. This is a consequence of limitations in retinal acuity, which is greatest within a narrow region of central vision (extending about 1° each side of fixation, corresponding roughly to the fovea) and declines sharply outside this region [59]. At a normal reading distance, about three or four letters will subtend about 1° of visual angle, so at most about six to eight letters can be seen clearly on each fixation. Saccadic eye movements, therefore, serve to shift the reader’s gaze along each line of text so that successive chunks of linguistic information (i.e., words) are processed in high acuity.

Given that skilled reading essentially involves integrating these snapshots of linguistic information, it is important to understand how much text can be processed on a single fixation. The area of text from which useful information can be acquired on each fixation is called *the perceptual span*. This has been widely investigated using gaze-contingent moving window paradigms ([60,61]; for a review, see [62]). In these, text is shown normally within a narrow region (window) around gaze and text outside the window is masked (e.g., by replacing letters in words with ‘x’s). This window moves in synchrony with the reader’s eye movements so that when the reader fixates a new location, text within the window at this new location is shown normally, and text at the previous fixation location is masked. This ensures only a small amount of text is seen normally on each fixation. These paradigms are implemented using high-speed computer programming and display screens with a fast refresh rate so that a participant’s phenomenological experience is that the windows move in perfect synchrony with their eyes. Moreover, by systematically varying the size of the window across an experiment, it is possible to investigate which size produces normal reading rates, following the logic that this window must encompass the perceptual span. Studies taking this approach show that skilled young adult readers (of English) primarily obtain useful information from an asymmetrical region extending about 14–15 letters to the right of fixation and 3–4 letters to the left ([60,61,63], but see [64,65]). Roughly speaking, this means they acquire information about the word they are fixating and about two words to its right (including information about the boundaries between words). The perceptual span can vary, however, as a function of reading expertise and the script that is being read [66,67,68,69,70,71,72,73,74,75,76]. For instance, span size is smaller for beginning and dyslexic readers compared to skilled adult readers, and for readers of dense scripts like Chinese compared to English.

Surprisingly little aging research has been conducted on the perceptual span, however, although non-reading studies show older adults process information outside of central vision less effectively than young adults [19,20]. Consistent with this more limited visual processing, several studies by Keith Rayner and his colleagues suggest that older adults acquire less information on each fixation compared to young adults. The first study to examine these effects, by Rayner, Castelhano, and Yang (2009) [77], used a standard gaze-contingent paradigm in which letters in words outside a moving window were masked by replacing them with ‘x’s. As is typical for studies using this paradigm, young adults read fastest when the fixated word and two words to the right were visible within the moving window. By comparison, older adults showed no difference in performance when the fixated word and either one or two words to the right were visible, suggesting they obtained less information about upcoming words. Moreover, unlike the young adults, the older adults were disrupted when the word immediately to the left of the fixated word was masked. Rayner et al. took this to show that the perceptual span is smaller and more symmetrical for older compared to younger adults, the implication being that older readers make less use of information about upcoming words.

Support for this came from a subsequent study by Rayner, Castelhano, and Yang (2010) [78], using the boundary paradigm. In this paradigm, an invisible boundary is placed immediately in front of a specific target word in each sentence. This word is masked prior to the reader’s gaze crossing the boundary, after which it is changed to normal. As with the moving window paradigm, these changes are made very fast, using high-speed computing and displays with fast refresh rates, and usually completed within the time it takes the reader to make a saccade. As a result, readers are generally unaware that a change has been made. Rayner et al. found that restricting the visibility of an upcoming word disrupted reading less for older than younger adults, again suggesting that older readers make less use of parafoveal (i.e., upcoming) information. Finally, Rayner, Yang Schuett, and Slattery (2014) [79] showed that older adults were significantly more impaired than younger adults when a moving window masked each fixated word. That is, as a reader moved their gaze along a line of the text, each word they looked at was masked while other text was shown normally. The greater difficulty this caused older adults was taken as further evidence that they use parafoveal information less effectively.

The overall picture from these studies is that the perceptual span is smaller for older than younger adult readers, quite possibly because of reduced parafoveal visual abilities or because higher attention demands associated with the processing of fixated words limit resources available for parafoveal processing. However, while these findings fit well with the notion that older readers process less information on each fixation, other studies are less supportive. First, Whitford and Titone (2016) [80] found no perceptual span differences for young and older adult bilinguals when both letters in words and the spaces between words were replaced by a dash (-). Second, Risse and Kliegl (2011) [81] provided evidence that older adults’ parafoveal processing may actually be relatively well-preserved. They used an adaptation of the boundary paradigm, in which the availability of the second word following a target word (i.e., word N+2) was manipulated. This was shown normally or masked so that it was not identifiable until the reader made a saccade that crossed an invisible boundary immediately following the target word. The key consideration was whether masking this word would disrupt reading. Risse and Kliegl found that it did, but to the same extent for young and older adults. This suggested both age groups could use parafoveal information from up to two words ahead of a fixated word equally effectively, inconsistent with the view that older readers acquire less parafoveal information on each fixation.

Paterson, McGowan, and Jordan (2013) [82,83] took a different approach to this problem and speculated that older readers might obtain different qualities of visual information from outside central vision, based on evidence that their sensitivity to fine visual detail is reduced, especially in peripheral vision [11,12,13,14]. They used a moving window paradigm in which the spatial frequency content of text outside each window was filtered. This ensured only either low, medium, or high spatial frequency content was available. Low spatial frequencies convey coarse-grain blurry information that lacks detail but provides visual cues to the overall shape and location of words. By comparison, high spatial frequencies provide sharp edge-like features for letters without their normal density, while the quality of visual information provided by medium spatial frequencies is somewhere in-between. The findings showed that reading times were closer to normal for young adults for text containing only high rather than low spatial frequencies, but closer to normal for older adults when spatial frequencies were low rather than high. The indication, therefore, was that older readers require primarily coarse-scale information about the shape and location of words from outside central vision. A subsequent study by Jordan, McGowan and Paterson (2014) [84] used a version of this paradigm in which spatial frequencies were filtered within the moving window and text outside was shown normally. This produced a similar pattern of effects, suggesting that older readers have reduced sensitivity to visual detail even within central (i.e., foveal) vision.

Such findings raise broader questions concerning sensitivity to different types of parafoveal (and perhaps even foveal) information by different groups of readers. One issue relates to the use of regular patterned masks in many moving window experiments, where letters in words are replaced by ‘x’s or dashes. The regular pattern created by these masks is usually apparent to participants. This may, therefore, serve as a distraction that disrupts normal reading performance rather than a baseline condition in which access to parafoveal information is restricted. As considerable evidence suggests older adults suffer more from distraction than young adults [85,86,87], they may be more affected by these masks. Consequently, while research on adult age differences in perceptual span effects currently is inconclusive, a way ahead may be to consider more carefully potential age differences in the effects of different types of foveal and parafoveal masks.

## 3. Aging and Mechanisms of Eye Movement Control

The basic characteristics of eye movements during reading are relatively well understood, certainly for skilled young adult readers of alphabetic scripts like English (for reviews, see [2,3]). Most saccades are forward-directed and traverse a distance of about 7 to 9 character spaces, while a small proportion (typically 10%–15%) are backward eye movements (called regressions) made to re-read text in response to some difficulty. Forward eye movements tend to be shorter and regressions more frequent when text is more difficult. Skilled readers also fixate most words at least once, with each fixation lasting between 100 and 500 ms and averaging about 250 ms. Readers sometimes skip words, however, on about 10%–20% of saccades, by moving their gaze past a word without fixating it.

Research also shows that skilled readers target their saccades towards upcoming words extremely accurately and that the landing positions of these saccades (i.e., the location of the resulting fixation) is systematically related to the physical length of a word. In particular, saccades tend to land at a so-called preferred viewing location (PVL) between the beginning and middle letters of a word [88,89]. This has been interpreted as evidence that readers target their saccades towards the word center but often undershoot this location due to oculomotor error [90]. Such measures have generally been taken from only one eye in experimental studies (typically the right eye or dominant eye). However, studies that have examined the coordination of the movements of the two eyes show that the two eyes fixate the same letter in a word on about 50% of fixations ([91,92,93], for a review see [94]). On other fixations, the eyes are crossed (so that the left eye fixates to the right of the right eye) or uncrossed (so that right and left eye fixations are divergent), although the relative preponderance of crossed and uncrossed fixations varies across studies and may depend on lighting conditions. Moreover, while the fixations made by the two eyes on average are only about 2 characters apart, this disparity has a high variance so that sometimes the fixations can be much further apart and even on different words.

Studies comparing the eye movements of older adult readers (typically 60+ years) and young adult controls (18–30 years) show that older adults make more and, on average, longer fixations and more regressions [64,80,82,83,84,95,96,97,98,99,100]. These findings suggest the older adults read more slowly and experience greater reading difficulty. More controversially, findings from numerous studies suggest older readers make longer forward saccades (on average) and skip words more frequently compared to young adults. This has been attributed to older adults adopting a qualitatively different reading strategy to compensate for greater reading difficulty [97]. In particular, according to this account, older adults employ a “risky” reading strategy in which they are more likely, compared to young adults, to infer the identities of upcoming words based on contextual knowledge and only partial parafoveal word information, with the result that they also skip these words more frequently. It also is argued that this risky reading strategy results in the more frequent misidentification of words and so may contribute to an increased rate of regressions by older readers.

Increased skipping and longer forward saccades are not always observed in aging studies, however, leading some researchers to question the evidential support for risky reading [100,101]. For instance, both Choi et al. (2007) [101] and Whitford and Titone (2017) [100] found that older participants in their experiments did not skip words more frequently than young adults. However, one possibility, also considered by these critics, is that the reading strategy used by the older adults may be sensitive to task demands and so vary as a function of text difficulty. Wotschack and Kliegl (2013) [102] found that age differences in word-skipping can be modulated by the frequency and difficulty of the comprehension questions that follow sentence displays on at least a proportion of trials in most eye movement studies. In particular, whereas older adults showed higher skipping compared to young adults when questions were easy and asked infrequently, this difference was reduced when questions were harder and asked more often. This may explain the absence of an age difference in word-skipping effects in the Choi et al. study [101], as it included comprehension questions after every trial. Moreover, the paradigm employed by Choi et al. involved making surreptitious changes to upcoming words and used stimuli containing deliberate misspellings, which also may have encouraged participants to read carefully. The Whitford and Titone (2017) study [100] examined paragraph reading, and, having read each paradigm, participants had to answer multiple open-ended comprehension questions. As the authors note, this may have imposed attentional and working memory demands that also encouraged the older adults to read very carefully.

These studies are valuable in highlighting the potential that older readers may adopt different strategies depending on task demands. It will, therefore, be important to establish what factors cause older readers to employ a particular reading strategy (and for discussion of effects of reading strategy and goals on eye movements see [44,103,104,105,106,107]). It will also be important to establish whether risky reading is used to compensate for slower processing in older age as Rayner et al. (2006) [97] proposed. This should include considering the consequences of risky reading for processes of word recognition during reading. According to the original account, older readers are more likely to misidentify words based on their contextual expectations, lexical knowledge and parafoveal cues to the identity of the next word. As parafoveal visual cues are likely to be degraded [18], it seems likely that older readers might misidentify a word due to its visual similarity to another word that better fits the context. However, only one study to date, by Warrington et al. (2008) [108], has examined this possibility. This used stimuli from an earlier study [109] in which target words (e.g., “brunch”) in sentences often had a lexical neighbor (i.e., a word sharing all but one of the same letters as the target, e.g., “branch” [110], that provided a similar or better fit to the sentence context. This neighbor word was also more commonly used in written language and so of higher lexical frequency. The original study showed that participants were more likely to make a regression to re-read the target word when it had a such a neighbor than when it did not, suggesting that readers often misidentify a word by temporarily mistaking it for its higher frequency neighbor (even though that neighbor does not appear in the text). The follow-up study by Warrington et al. [108] found young and older adults were both likely to misidentify a target word as its neighbor when this better fit the context, and so made more regressions to re-read the target. However, post hoc analyses (not reported in the paper) suggested this effect was greater for the older adults when the target and its neighbor were visually similar (e.g., “brunch” and “branch”) rather than visually dissimilar (e.g., “story” and “stork”). Accordingly, a potentially fruitful approach to exploring the consequences of risky reading might involve investigating misreading errors by older adults.

Finally, in this section, we consider whether eye movement control and saccade-targeting are poorer for older readers and whether this might contribute to the difficulty they experience. In the Introduction, we reviewed evidence that eye movements might be slower in older age due to impaired motor and attentional control, but that accuracy in saccade-targeting was unlikely to be affected by age. Studies to date have not examined aging effects on eye movement dynamics in reading. Moreover, only a couple of studies have examined saccade-targeting. Consistent with evidence from non-reading tasks [23,25,30], this appears to be preserved. For instance, Rayner et al. (2006) [97] examined the location of initial fixations on words during reading and observed a very similar distribution for young and older adults, consistent with both age groups preferentially fixating near the PVL. A subsequent study by Paterson et al. (2013) [96] found that the pattern of landing positions for short (5-letter) and long (10-letter) words did not differ appreciably for young and older adults. This study additionally examined the coordination of binocular eye movements. This was motivated by evidence that reading difficulty is likely to produce larger fixation disparities [91,111], and other non-reading studies, suggesting older adults naturally produce larger disparities [112,113]. However, the results showed no age differences. Moreover, the magnitude of the disparity was similar to previous research, averaging about two letter spaces apart [93], with a similar predominance of crossed over uncrossed fixations. Consequently, while older adults are slower and experience greater reading difficulty, basic mechanisms of oculomotor control for reading appear to be preserved in older age.

In addition to these studies, two experiments have investigated the effects of removing or filling the spaces between words in sentences. Text in most alphabetic languages customarily includes spaces between words. By helping to demarcate word boundaries, these can aid the processing of words by reducing visual crowding and lateral masking (interference from flanking letters) of exterior letters in words [114]. Moreover, interword spaces provide useful coarse-scale (i.e., low spatial frequency) information about the location and physical extent of words and so can aid eye-guidance [115]. Removing or replacing these spaces disrupts normal reading (for languages that customarily include spaces [116,117,118,119,120]). Moreover, several studies show that this impairs word identification by producing larger than normal word frequency effects. An important question, therefore, is whether older readers suffer more when inter-word spaces are removed.

Rayner, Yang, Schuett, and Slattery (2013) [120] showed this is the case. Compared to young adults, the older adults in their study had much larger increases in sentence reading times when spaces were removed. They assessed effects on the lexical processing of words by examining the influence of this manipulation on the word frequency effect (i.e., the processing advantage for words that have a high frequency of written usage). The rationale for this approach was that if removing spacing disrupted word identification processes, this should impact more on low compared to high frequency words and so produce a larger word frequency effect. This is what was observed. However, the increased size of the word frequency effect was comparable for young and older adults, suggesting no age difference in the effects of spacing on word identification. A subsequent study by McGowan, Jordan and Paterson (2014) [64] confirmed these findings but also examined conditions in which spaces between words were filled using a closed square (■) or an open square (□). Disruption to normal reading was greater for older adults when an open rather than a closed square was used. McGowan et al. explained this in terms of the salience of these squares as cues to word boundaries. The closed squares provide a low spatial-frequency cue to word boundaries. By comparison, the open squares were composed of high spatial-frequency visual components (i.e., horizontal and vertical lines) similar to letter features. Older adults appeared, therefore, to have particular difficulty when word boundaries were delineated by a high-spatial frequency cue, and less difficulty when these were demarcated by low spatial-frequency cues, quite possibly due to reductions in sensitivity to higher spatial frequencies. Indeed, it seems likely that older readers had difficulty discriminating between the visual features of the open circles and those of adjacent letters in words, impairing their ability to delineate words in the text.

## 4. Aging and Eye Movements in Alphabetic Reading

A key research finding is that eye movements are under cognitive control, meaning that decisions about when and where the eyes move are governed by factors influencing how easily words can be identified. Research with alphabetic languages suggests that chief amongst these factors are the length of words, their familiarity to the reader (generally operationalized as the frequency of lexical usage), and their predictability from prior context (for reviews, see [2,3]). Consistent with this account, numerous studies show that words which are shorter, higher in lexical frequency, or more predictable have lower fixation probabilities (and so are more likely to be skipped) and shorter reading times compared to words that are longer, of lower frequency, or less predictable [88,95,96,97,101,121,122,123,124,125,126,127,128,129,130]. Such findings have been important in furthering our understanding of mechanisms of eye movement control in reading and led to the development of sophisticated computational models, such as E-Z Reader [131,132] and SWIFT [133].

Despite substantial evidence that older adults experience greater reading difficulty (see the previous section), few studies have investigated aging effects on the cognitive control of eye movements during reading. Both a corpus-based analysis [95] and an experimental study [96] suggest that aging and word length effects are additive so that longer words are not especially difficult for older readers to process. Other studies have examined age differences in the word frequency effect when other factors, including word length and predictability, are controlled. As noted above, higher frequency words receive shorter reading times and are more likely to be skipped. However, older adults typically produce larger word frequency effects than young adults by fixating for longer on words that are of lower rather than higher frequency [64,95,97,100,120]. This is consistent with older adults having greater difficulty identifying words due to slower lexical processing (due either to cognitive decline or as a consequence of increased lexical items in the mental lexical [42,43]).

These effects have been simulated computationally (in E-Z Reader and SWIFT) for alphabetic languages by limiting the area of text that is sampled on each fixation and slowing lexical processing [97,134]. These simulations capture the greater difficulty older readers experience when identifying lower frequency words. Simulations based on the E-Reader model additionally are in line with the “risky reading hypothesis” proposed by Rayner et al. (2006) [97]. This predicts that older readers skip words more often and make generally longer forward eye movements. Within the model, this is achieved by changing parameters associated with the role of contextual prediction in helping a reader to anticipate the next word in a sentence. This makes sense theoretically as it would be plausible for older readers to make greater use of context to compensate for slower lexical processing [97,135]. Such effects are also consistent with findings from research on spoken and written word recognition, suggesting differential use of context by adult age groups and, in particular, that older readers benefit more from supportive contexts when input is degraded [50,52,53,136,137,138,139].

The simulations predict that older readers are more likely than young adults to skip words that can be predicted from the prior context and also that reading times for these words will be shorter. However, evidence for an adult age difference in word predictability effects has proven elusive. A corpus study [95] showed that high word predictability resulted in faster reading by both young and older adults. However, this was realized differently for the two age groups, by increasing word-skipping by the young adults and decreasing the older adults’ probability of making multiple fixations on words without affecting their word-skipping rates. Crucially, however, even the experiment that Rayner et al. (2006) [97] reported alongside their simulations provided little evidence of a larger word predictability effect for older readers. This study experimentally manipulated the cloze predictability of target words in sentences. This involved creating contexts in containing a target word that was either highly predictable or one that was less predictable, but where both words were plausible continuations. For instance, in a sentence like “Harriet sang while my brother played piano /flute for my birthday”, “piano” is a more likely continuation than “flute”, although both words make sense in the context. Rayner et al. established the greater predictability of one word over another using a task in which participants (who do not also take part in the experiment) provide a written continuation for the beginning fragment of a sentence (e.g., “Harriet sang while my brother played…”) and how frequently different words are used as completions is assessed. Sentences containing words that were highly predictable or less predictable continuations were then used as stimuli in the experiment. The results showed that higher word predictability increased skipping rates only marginally more for older compared to younger adults, and shortened reading times similarly for both age groups. There was therefore little evidence that older readers made greater use of predictability to skip words or speed the processing of words they fixated. However, as this study included a manipulation of font difficulty, participants may have had difficulty encoding text on some trials and so read more carefully than normal, which may explain why larger benefits of word predictability were not observed.

More propitiously, a recent experiment by Steen-Baker et al. (2017) [140] found a positive relationship between age and effects of the cloze predictability of a sentence-final word on reading times, such that the decrease in reading times produced by higher predictability was larger for older adults. However, this study was not informative about predictability effects on word-skipping as the target word was always the final word in a sentence (and so could not be skipped). Moreover, the reading time measure they used included time spent re-reading the sentence after encountering the target word and so will have captured sentence-wrap up effects as well as effects of predictability on the processing of the target word. Only one eye movement study to date, by Choi et al. (2017) [101], provides clear evidence for an adult age difference in predictability effects during the initial processing of words, although this was observed only in reading times for words without affecting word-skipping. The study showed that predictability speeded the recognition of words when these were fixated, with no evidence that it facilitated word-skipping. However, several other features of the study may have mitigated against observing such effects. In particular, the task used by Choi et al. included surreptitious changes to target words, deliberate misspellings, and included a comprehension question after each trial (see the previous section), which may have encouraged the older adults to read more carefully and skip words less often than normal.

The research reviewed above addressed key questions about aging effects on mechanisms of eye movement control during reading. This has developed our understanding of some influences of aging, especially in relation to effects of word length and word frequency, although even here relatively few studies have been conducted. Of greater concern is the paucity of studies focused on the claim that older readers make greater use of context to compensate for slower lexical processing. Only a few studies have addressed this question by investigating effects of word predictability and, while there is some indication of such an effect, further work is required to understand it more fully. It will be especially important to establish if, compared to younger adults, older adult readers make greater use of predictability to infer the identities of upcoming words and so skip them. However, more generally, it will be valuable to understand how older adults make use of contextual information and the extent to which their greater knowledge of words and experience of reading might lead them to use prediction or anticipation to a greater extent.

## 5. Aging and Eye Movement Control in Chinese Reading

Research on aging effects in reading have been conducted predominately in alphabetic languages in which words are created from letters, and words in sentences are clearly delineated using spaces. With this section, we review a relatively small number of studies conducted in Chinese, a non-alphabetic language with visual and linguistic characteristics very different to those of alphabetic languages like English and German. Investigating effects in this language is important for many reasons, including that Chinese is the most widely used writing system worldwide, and that China has the most rapidly aging population. Moreover, such investigations allow us to assess whether aging effects are similar cross-linguistically or reflect the specific visual and cognitive demands of the writing system.

Text in Chinese is composed of box-like logograms, called characters, which can differ considerably in their number of component strokes (lines, dashes), while always occupying the same square area of space [141,142]. For instance, simple characters may be created from a single stroke (e.g., 一 [“yi”], meaning “one”) while more complex characters contain upwards of 20 strokes (such as 罐 [“guan”], meaning “pot”). Chinese characters can, therefore, vary substantially in visual complexity, and evidence from non-reading studies suggests such effects may be an important source of perceptual difficulty. For instance, assessments of character legibility show that higher acuity is required to recognize characters with more strokes [143,144]. Moreover, research investigating the visual span for Chinese characters (i.e., how many characters can be recognized on a single glance without moving the eyes) shows span size is smaller for more complex characters [145]. This effect appears to be even greater for older adults [146], suggesting they may recognize fewer characters on each fixational pause during reading, although published research to date has not investigated aging effects on the perceptual span in Chinese reading. Finally, while some characters can function as a word, most Chinese comprise two or more characters. Indeed, according to the Lexicon of Common Words in Contemporary Chinese (2008) [147], only 6% are one-character words, 72% two characters, 12% three characters, and the remainder mostly four characters. However, written Chinese does not include spaces or other cues to word boundaries. An important task when reading Chinese, therefore, is to segment this naturally unspaced text into words (for further discussion of the specific visual and cognitive demands of the Chinese writing system see [148,149]).

Despite these differences, research suggests the same fundamental variables are important in determining when and where the eyes move in Chinese reading. In particular, research with young adults shows that, as with alphabetic languages, reading times are faster and skipping rates higher for words that are shorter (and so composed of fewer characters), of higher frequency, or more predictable from context [150,151,152,153,154,155,156,157,158]. This suggests cross-linguistic similarity in basic mechanisms of eye movement control. These similarities have also enabled researchers to develop computational models of eye movement control in Chinese reading following the same principles used to develop models for alphabetic languages [159]. It has also led researchers to consider whether older readers produce patterns of age-related reading difficulty in Chinese reading similar to those observed with alphabetic languages. Relatively few such studies have been conducted to date. However, the effects show clearly that older readers experience considerably greater reading difficulty. Compared to young adults, they read much more slowly (often almost twice as slow) and make more and longer fixations and regressions [154,155,158,160,161]. However, by contrast with evidence from alphabetic languages, there is no indication that older Chinese readers use a more risky reading strategy to compensate for this slower reading. Compared to young adults, they appear to skip words infrequently, and their forward saccades are either shorter or of similar length, consistent with more careful reading. Therefore, while age-related reading difficulty is observed cross-linguistically, its consequences appear to differ depending on the writing system.

Studies that have examined aging effects on eye movements in Chinese reading also show that word frequency effects are larger for older than younger adults, suggesting that, as with alphabetic languages, older readers are slower to recognize words [154,155]. Others show that the visual complexity of Chinese characters has a larger effect on reading times by older adults than younger adults [159,162], indicating that this may be an important source of age-related reading difficulty. Only one study to date has examined adult age differences in word length effects [152], although this may be especially important in helping to understand the nature of the difficulties experienced by older Chinese readers. This study examined eye movements for two- and four-character words matched for frequency and embedded in identical sentence contexts. By contrast with additive effects of aging and word length in research with alphabetic languages [96], the effects of these variables produced an interaction in reading times, due to older adults having greater difficulty for long compared to short words. Why this interaction effect is observed became clearer once the landing positions of fixations was examined. Previous research on saccade landing positions in Chinese reading presents a complex picture in which the pattern differs for words receiving either only one fixation or multiple fixations during their initial reading [151,162]. Landing positions on words receiving only one fixation tended to be close to the center of short and long words. By comparison, landing positions on words receiving multiple fixations tended to land on the first character of words regardless of word length. This pattern has been interpreted in different ways. Yan et al. (2010) [162] proposed that readers select either the beginning or center of words as a saccade target depending on whether they can obtain parafoveal cues to word length. That is, if information about the length of the upcoming word is available, the reader will target their next saccade towards the center of that word. However, if this information is unavailable, the reader will employ a more cautious strategy that targets their next saccade towards the first character of the next word. By contrast with this account, X. Li et al. (2011) [151] argued that the effects are not due to use of parafoveal word length cues. They argued instead that the effects reflect the fact that words can be recognized more quickly, and so are less likely to be re-fixated, when a saccade just happens to land at an optimal intra-word location (i.e., word center). That is, readers are more likely to make only one fixation on a word when the initial saccade lands fortuitously at its center, and more likely to make multiple fixations on words when their initial saccade lands at a less optimal location. X. Li et al. proposed that parafoveal processing is character- rather than word-based in Chinese reading and that readers achieve processing efficiency by estimating how many upcoming characters they can identify on each fixation and targeting their next saccade to the right of these characters (see also [163,164]). Crucially, however, while the underlying mechanisms differ, both accounts highlight the importance of parafoveal processing for eye guidance in Chinese reading.

The young adults in the study by S. Li et al. (2018) [152] produced the same pattern of landing position effects as these previous studies. The older adults, however, made fewer single fixations on words, especially longer words, and their saccades were much more likely to land towards the beginning of the words. An exploratory analysis of subsequent fixations additionally showed that older adults were more likely to make a sequence of fixations that landed successively on each character in a word. This was taken as evidence that older readers employ a cautious strategy in which they inspect words character by character. This may explain why older Chinese readers exhibit a pattern of age-related reading difficulty different from that of older readers of alphabetic languages. It may also result from specific difficulties experienced by older Chinese readers when processing upcoming characters or segmenting unspaced text. However, research has yet to address these questions and so an important way forward will be to investigate age differences in parafoveal processing and word segmentation in Chinese reading. A final consideration that also has received little attention to date concerns whether older Chinese readers make greater use of word predictability to offset the difficulties they experience. Only one study has been reported to date [161]. This showed word predictability has a larger facilitatory influence on reading times for older adults but no corresponding effect on word-skipping. Strikingly, such effects may provide the strongest evidence to date (in alphabetic or non-alphabetic languages) for the greater use of contextual knowledge by older readers.

In sum, studies reviewed in this section show very clearly that investigations of aging effects in Chinese are important in demonstrating that age-related reading difficulty is experienced cross-linguistically but may reflect the specific visual and cognitive demands of the writing system. The review highlights that further work is necessary to fully understand the nature of these difficulties. In particular, the evidence that older readers have greater difficulty processing words due to the visual complexity of characters, and make greater use of context, requires more detailed investigation. Moreover, the indication that older readers have specific difficulties parafoveal processing and segmenting words in unspaced text has yet to be explored but likely to be central to our understanding of aging effects on Chinese reading.

## 6. Conclusions

It should be clear from this review that normative aging has pervasive effects on eye movements in reading. These are a consequence of changes in visual, attentional, and cognitive abilities that occur naturally in older adulthood. They also appear to depend on the characteristics of the writing system and differ subtly for readers of alphabetic languages compared to non-alphabetic languages like Chinese. For the most part, the age-related changes we observe are not catastrophic although clearly likely to affect the efficiency and ease of reading by older people, which will have practical implications and impact on their quality of life. However, it is notable that most research has been conducted with healthy, active, and well-educated older people, and it will be important to more fully understand effects of aging across a spectrum of older people, as well in relation to age-related visual impairment (e.g., macular degeneration) and neurodegenerative disease (e.g., Alzheimer’s disease). Indeed, what is clearest from the review is that current understanding of aging effects is limited, and more work is required to fully understand the difficulties that older readers experience.
